# Molecular Signaling of Progesterone, Growth Hormone, Wnt, and HER in Mammary Glands of Dogs, Rodents, and Humans: New Treatment Target Identification

**DOI:** 10.3389/fvets.2017.00053

**Published:** 2017-04-13

**Authors:** Elpetra P. M. Timmermans-Sprang, Ana Gracanin, Jan A. Mol

**Affiliations:** ^1^Department of Clinical Sciences of Companion Animals, Utrecht University, Utrecht, Netherlands

**Keywords:** progesterone, growth hormone, Wnt signaling, HER, canine mammary cancer

## Abstract

Mammary tumors are the most common form of neoplasia in the bitch. Female dogs are protected when they are spayed before the first estrus cycle, but this effect readily disappears and is already absent when dogs are spayed after the second heat. As the ovaries are removed during spaying, ovarian steroids are assumed to play an essential role in tumor development. The sensitivity toward tumor development is already present during early life, which may be caused by early mutations in stem cells during the first estrus cycles. Later on in life, tumors arise that are mostly steroid-receptor positive, although a small subset of tumors overexpressing human epidermal growth factor 2 (HER2) and some lacking estrogen receptor, progesterone receptor (PR), and HER2 (triple negative) are present, as is the situation in humans. Progesterone (P_4_), acting through PR, is the major steroid involved in outgrowth of mammary tissue. PRs are expressed in two forms, the progesterone receptor A (PRA) and progesterone receptor B (PRB) isoforms derived from splice variants from a single gene. The dog and the whole family of canids have only a functional PRA isoform, whereas the PRB isoform, if expressed at all, is devoid of intrinsic biological activity. In human breast cancer, overexpression of the PRA isoform is related to more aggressive carcinomas making the dog a unique model to study PRA-related mammary cancer. Administration of P_4_ to adult dogs results in local mammary expression of growth hormone (GH) and wing less-type mouse mammary tumor virus integration site family 4 (Wnt4). Both proteins play a role in activation of mammary stem cells. In this review, we summarize what is known on P_4_, GH, and Wnt signaling in canine mammary cancer, how the family of HER receptors could interact with this signaling, and what this means for comparative and translational oncological aspects of human breast cancer development.

## Introduction

Mammary tumors are the most common neoplasms in intact bitches with an estimated life-time risk of 1:4. About 50% of these tumors are malignant and one-third of these may form life-threatening distant metastases. Breast cancer is also a very common disease in humans with a life-time risk of about 1:8 (1).

Hierarchical cell organization of the mammary gland has recently become more clear in humans and mice and is linked to the heterogeneity of the mammary epithelium, with an outer and inner layer of luminal cells. Both layers have different epithelial cells with differential characteristics. In the outer basal layer of contractile myoepithelial cells, the mammary stem cells (MaSCs) reside, whereas in the layer of ductal and alveolar epithelial luminal cells, progenitor cells are found (2–4).

The main problem in breast cancer treatment is the recurrence of tumor growth and metastases. In both cases, cancer stem cells (CSCs) are thought to play an important role. However, the stem cell for the mammary gland has not yet been identified. The current paradigm is that a common stem cell gives rise to progenitor cells that are intermediates in the lineages of myoepithelial, and epithelial duct or lobular cells (5). In dogs, a subset of mammary carcinomas present as simple carcinomas that may be derived from mutated epithelial progenitor cells and are comparable to the most common form of human breast cancer, the ductal carcinomas. However, more often than in humans, dogs may also present with complex carcinomas that contain various differentiation pathways within a single affected mammary gland. These tumors may be derived from mutations in the most basic and early form of stem cells. The existence of the MaSC and the presence of various forms of progenitors may in part explain the heterogeneity of mammary carcinomas (6).

## Hormone-Dependent Mammary Gland Development, Including Normal Roles of Growth Hormone (GH), Wnt, and HER

During embryogenesis, mammary gland development starts with the formation of a mammary placode, and subsequently a mammary bud. The functional development and differentiation of the mammary gland occurs, however, mainly postnatally under hormonal control and is coordinated with further reproductive development. Puberty starts with a trigger from estrogen (E_2_) and local growth factors to elongate the simple ductal tree by stimulating cell proliferation in the terminal end buds (7–9). Subsequently, fluctuating levels of progesterone (P_4_) stimulate the process of side branching and development of alveolar buds. During pregnancy, in response to P_4_ and prolactin (PRL), these alveolar buds can then differentiate into functional milk producing units, alveoli (7–9). P_4_ is thought to induce these changes in the mammary gland in a paracrine manner by acting on the progesterone receptor (PR)-expressing ductal epithelial cells, to stimulate the expression of growth factors that evoke proliferation of the neighboring PR-negative cells (10). These putative paracrine factors involve GH *via* signal transducer of activator of transcription (Stat3) (11) and of Janus kinase 2 (Jak2)/Stat5 (12), Wnt, and receptor activator of nuclear factor kappa B ligand (RANKL) (13–16).

Progesterone, together with E_2_, plays a central role in the outgrowth of the mammary gland by stimulating side branching of the mammary ductal tree during puberty and alveologenesis upon pregnancy. Upon P_4_ activation, PR-positive epithelial cells secrete GH and Wnt4 that act on MaSCs. In mammary cancer, tumor cells with stem cell properties, such as phenotypical epithelial mesenchymal transition (EMT) and elevated activity of the canonical Wnt pathway, play an important role in regrowth and metastasis (17, 18). In both humans and dogs, most mammary carcinomas are initially hormone dependent [i.e., express receptors for P_4_ (PR) and E_2_ (ER)]. The remaining tumors are often categorized as human epidermal growth factor 2 (HER2) positive (overexpressing HER2) or as triple-negative breast cancer (TNBC) (i.e., devoid of PR and ER and no overexpression of HER2) (1, 5, 7–9, 19).

## Goal of Review

Both human and canine mammary carcinomas show involvement of common pathways in mammary cell proliferation and migration, such as Wnt and phosphatidyl-3-kinase (PI3K) signaling (1, 19). An important aspect is the hormonal dependence in most human and canine mammary carcinomas and the role of P_4_. This underscores the relevance of research on canine mammary cancer for both human and veterinary medicine and is a clear example of the one-health/one-medicine principle. Commonly used rodent models develop mammary carcinomas that are either not hormone dependent or do not metastasize as seen in mice and rats. This raises the question of whether the study of canine mammary carcinomas is a valid or even a better model for human breast cancer in comparison to rodents.

This review focuses on what is known of P_4_, GH, and Wnt signaling in canine mammary carcinomas in relation to what is known in other species, especially human breast cancer. In addition to the many similarities, the differences will also be discussed.

## Progesterone

The central role of P_4_ signaling in breast cancer development gained renewed interest after the large hormone-replacement study of the World Health Initiative in 2003 (20). Since 1986, it has been known that ovarian steroids play an important role in the carcinogenesis of the mammary gland (21). Since then, elaborate research has been done predominantly on the role of E_2_. The tumorigenic role of P_4_ has long been underestimated (22) due to the more widespread use of synthetic progestins as inhibitors of tumor growth, even though this effect has been attributed to androgenic side effects since 1977 (23). In relation to reproductive physiology, P_4_ can have both a protective role or can be a risk factor for breast cancer. The protective role for P_4_ is seen in women who have an early full-term pregnancy and lactation. Risk factors associated with increased exposure to P_4_ are a prolonged interval between menarche and age of first childbirth, older age at menopause, early menarche, late menopause, and shorter menstrual cycles (24–27). Some of these factors influence development of a special subtype of breast cancer, for instance, lactation is correlated with TNBC and hormone responsive (HR^+^) tumors are associated with the length of the period between menarche and first childbirth (25, 26).

An important turning point for the role of P_4_ came from hormone-replacement studies that showed an increased risk for breast cancer development in women that received (conjugated equine) E_2_ plus a progestin [medroxyprogesterone acetate (MPA)] (28) compared to the E_2_ only group, in which there was a protective effect against breast cancer (20, 29). Similarly, in ACI rats that easily develop mammary cancer upon exposure to high dose E_2_, P_4_ was shown to be important for hormone-dependent mammary carcinogenesis (30). P_4_ signaling, therefore, clearly plays a role in breast cancer development, but the challenges are to define its exact role.

### PR Signaling

Progesterone signals *via* the PR. The PR is expressed as two isoforms, progesterone receptor A (PRA) and progesterone receptor B (PRB). Both isoforms are derived from a single gene but regulated by two distinct promoters. In humans, E_2_ stimulates expression of both PRA and PRB mRNA (31). This is in contrast with ovariectomized mice where PRA expression is stimulated by E_2_ and inhibited by P_4_. PRB levels are not affected by E_2_ alone, but are stimulated by prolonged treatment with P_4_ or by P_4_ in combination with E_2_ (32). Accordingly, in mice, PRA is the main PR isoform expressed during pre-pubertal stages and in adult virgins, while PRB expression increases only during pregnancy (33). Therefore, it has been suggested that in the mouse, the initial proliferative response of the mammary epithelium to P_4_, leading to side branching is mediated by PRA, while PRB is needed for a proper lobular alveolar development during pregnancy (32). By contrast, in the normal human breast, both PRA and PRB are coexpressed in the same cells implying species-specific regulation of the isoforms (34). The PR isoforms have isoform-specific transcriptional activities on P_4_-responsive gene promoters, resulting in a distinct target gene profile (35). PRs can activate gene transcription in multiple ways and direct binding to progesterone response elements (PREs) a process usually referred to as classical PR signaling. PR can also tether to other transcription factors, such as Stat5 in the regulation of RANKL expression. Finally, PR can mediate so-called “non-genomic” cytoplasmic signaling through interaction with ERα, thereby activating the Rous sarcoma proto-oncogene/(Ras–Raf–MEK–ERK) cSrc/ERK pathway (36).

**Figure 1:**
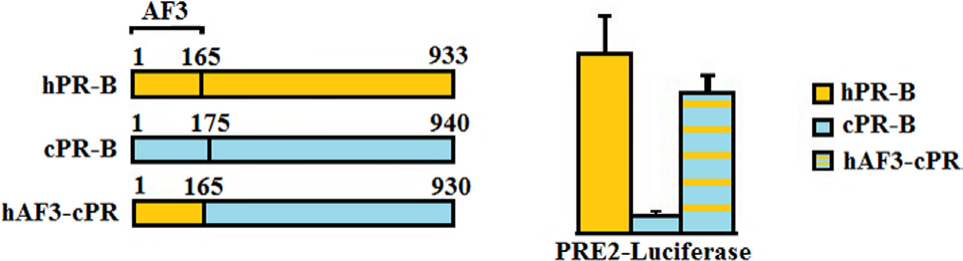
**Comparison of the progesterone receptor-B variant of human (hPR-B) and canine (cPRB) species (left panel)**. The PR-B variant is encoded by the same gene as for the PR-A variant but has, due to an alternative start of translation, an amino-terminal elongation known as activation function domain-3 (AF3). Measurement of transactivation potential, using a PR-sensitive luciferase-reporter construct showed that the activity of the progesterone-stimulated cPRB is almost absent in comparison to the hPR-B (right panel). Replacement of the AF3 domain of humans into the canine sequence almost completely restores the cPRB activity (41).

In classical signaling, both PR isoforms function as ligand-induced transcription factors and contain distinct activation function (AF) domains essential for their transcriptional activity. Two activation domains are common to both PRA and PRB, AF1 and AF2. PRB, however, has an additional activation function domain-3 (AF3) domain localized within the PRB specific N-terminus (37) making it a stronger transcriptional activator than PRA (Figure 1) (38). Sequence motifs essential for AF3 domain function were shown to be highly conserved across mammalian species (39, 40). We have compared the activities of canine PR (cPR) isoforms to human isoforms (hPR) using luciferase constructs containing classical PREs, mouse mammary tumor virus (MMTV)-luciferase, and PRE2-luciferase. We have shown that canine PRA has an expected hPRA-comparable transcriptional activity, whereas canine PRB (cPRB) has low to absent transactivation potential. No differences were found regardless of background cell type such as Chinese hamster ovary cells, canine mammary cells, or human T47D cells in which the endogenous PR was knocked out. The transactivation potential of cPRB could be restored by replacing the cPRB specific N-terminus with the human sequence in a human B-upstream segment (hBUScPRB) chimera. Next, we made canine mammary cell lines with a doxycycline (dox)-inducible expression of cPRB, hPRB, or the hBUScPRB chimera. Transactivation potential on endogenous target genes was then assessed by gene profiling using canine cDNA microarray. In the absence of dox, no effect of P_4_ incubation was seen, excluding signaling through P_4_ membrane receptors. Only the combination of dox plus P_4_ changed the expression of over 600 genes, both in hPRB- and hBUScPRB-expressing cell lines. Only a minority of these genes were influenced by cPRB (41, 42). These results indicate a very limited transactivation potential of cPRB on endogenous genes, thereby questioning its role in mammary gland development and carcinogenesis.

### PR Signaling in Mammary Cancer

Although the PRA and PRB isoforms are usually equally present in epithelial cells of the human mammary gland, in advanced breast cancer, a predominance of PRA is common, indicating that PRB has a protective function. Patients with PRA-rich tumors or tumors with a high PRA:PRB ratio have a much faster recurrence than patients with PRB-rich tumors (43, 44). Predominance of PRA is especially evident in ductal carcinoma *in situ* (DCIS) and invasive breast lesions. It has been suggested that P_4_ may also lead to transition of tumors from a luminal toward a basal phenotype (45). Germ line mutations in the genes breast cancer 1 (BRCA1) or breast cancer 2 are associated with a predominance of PRA expression (46). BRCA1 physically interacts with PR and inhibits its activity, in part, by preventing binding of the PR to the PRE and promoting the formation of a corepressor complex (47). Because this activity is lost in the context of BRCA mutant proteins, anti-progestins are recommended for tumor prophylaxis in BRCA mutation carriers (48). Mutation of BRCA1 results, moreover, in stabilization of the PR due to the loss of BRCA1-mediated PR ubiquitination and subsequent degradation (49). Interestingly, loss of BRCA1 also results in increased epidermal growth factor 1 (HER1) expression (50). We hypothesize that the combination of stabilization or enhanced PR expression and active HER1 signaling may stimulate phosphorylation of the PR by mitogen-activated protein kinase (MAPK) and specifically stimulate this mode of P_4_ signaling.

**Figure 2:**
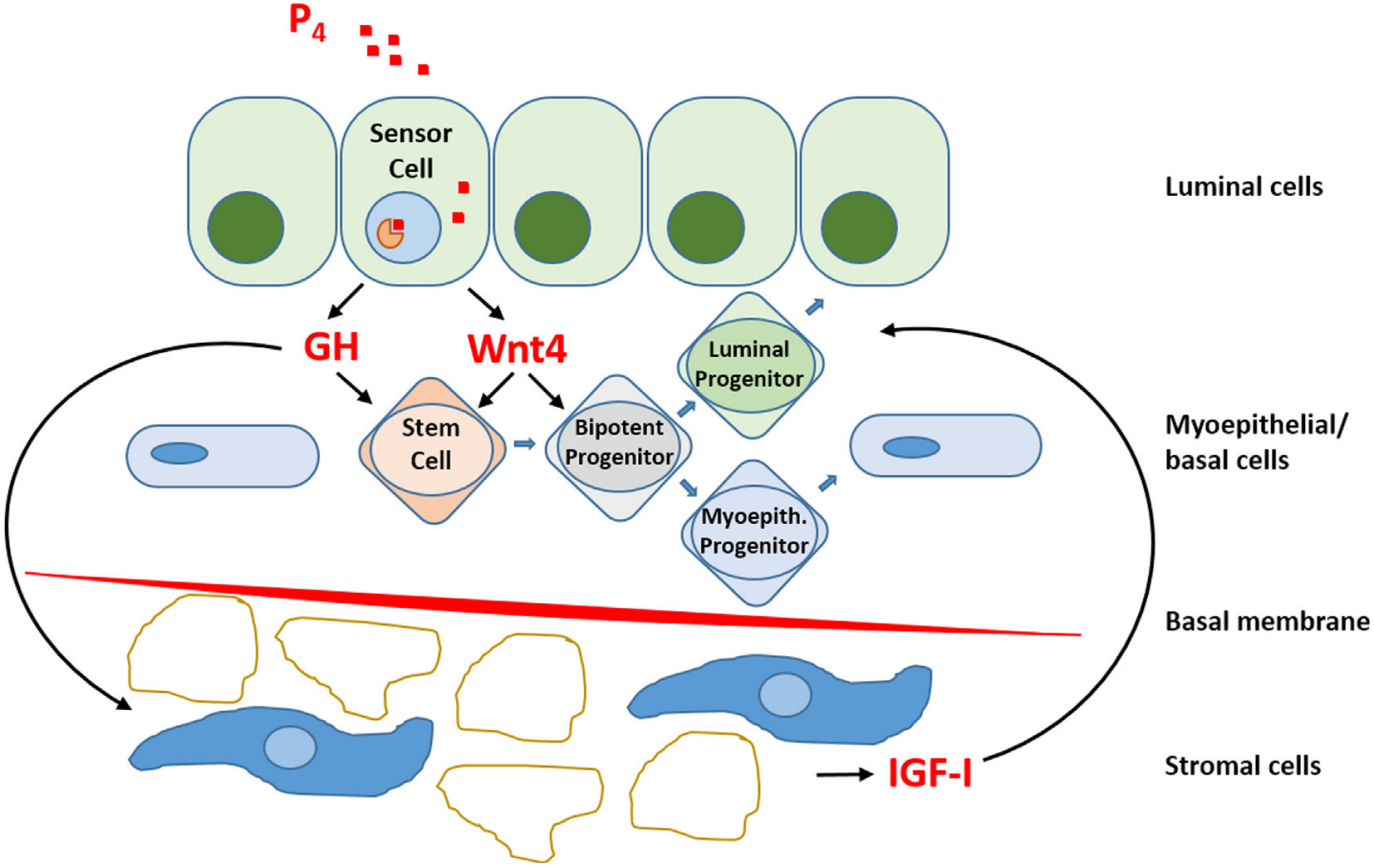
**Schematic representation of progesterone (P_4_) signaling within the mammary gland**. So-called “sensor cells” within the luminal epithelium contain progesterone receptors (PRs) that upon exposure to P_4_ stimulate the production and release of growth hormone (GH) and Wnt4. The local mammary production of GH has both a direct effect on GH receptor (GHR) containing stem cells and an indirect effect by stimulating the production and release of insulin-like growth factor-I (IGF-I) by GHR-containing cells in the stromal compartment. IGF-I stimulates further clonal expansion of activated cells. P_4_ also stimulates Wnt4 release. The Wnt pathway is essential for stem cell maintenance and activation of stem cells to form progenitor cells. Within the mammary gland, bipotent progenitor cells differentiate into progenitor cells specific for either luminal epithelial or myoepithelial cells.

Within the luminal epithelium of the mammary gland PR-positive cells act as sensors for circulating P_4_ concentrations (Figure 2). Upon P_4_ exposure, these cells secrete growth factors (RANKL, Wnt) that may stimulate recruitment and differentiation of stem cells (51). P_4_ thus induces adult MaSC expansion (52) in mice, and this is also hypothesized to be a major site of carcinogenesis within the human breast (53). As well as an increase in MaSC, P_4_ was also suggested to act directly on PR-positive cells and convert them to a hormone-receptor negative, more stem-like state (54). Cluster of differentiation (CD) CD44^High^CD24^Low^ cells have been reported as breast CSCs since Al-Haij showed that these cells can form tumors in mice (55). In humans, CD44^High^CD24^Low^ cells seem to have a higher tumorigenic capacity (56) and even in dogs these cells have a tumor-initiating capacity (57) and stem/progenitor cell properties (58). Cell markers for stem cells and progenitors in the human and murine mammary gland (59, 60) are summarized (Table 1).

**Table 1:** **Cell markers for stem cells and progenitors in the human and murine mammary gland (59, 60)**.

Cells	Human markers	Mouse markers
Stem	CD10^−^, CD24^−^, CD44^+^, ALDH^+^	Lin^−^, CD24^+^, CD29^++^, CD49f^++^, Axin2^+^
Bipotent progenitor	EpCAM^+^, CD49f^+^, CD133^−^, MUC1^−^, CD10^+^, THY^+^	
Myoepithelial/basal progenitor	Lin^−^, EpCAM^−^, CD49f^++^	CD45^−^, CD24^+^, CD49f^−^
Luminal progenitor	Lin^−^, EpCAM^+^, CD49f^+^, ALDH^+^, HER3^+^	Lin^−^, CD24^++^, CD29^+^, CD61^−^, Axin2^+^
Mature luminal	EpCAM^+^, CD49f^−^, CD133^+^, MUC1^+^, CD10^−^, THY^−^	Lin^−^, EpCAM^++^, CD49f^−^

These stem cells and progenitor cells are necessary in the mammary gland to ensure proper long-term maintenance of mammary tissue structure and function during puberty, pregnancy, and lactation, which may occur multiple times during the reproductive lifespan of an animal (60). Mouse MaSCs have the ability to resist anoikis and form floating colonies, the so-called mammospheres. Progenitor cells from the inner layer, the luminal cells, have high proliferative potential in colony-forming cell (CFC) assays (61, 62) and also have a high level of PR in humans (63). These high-level PR cells occur in poorly developed lobules of the mammary gland and can be characterized by lineage (Lin^−^), epithelial cell adhesion molecule (EpCam^+^), CD49f^+^, aldehyde dehydrogenase 1 (ALDH^+^), and human epidermal growth factor 3 (HER3^+^) (59). ALDH1 is associated with stem/progenitor cell properties in human mammary epithelial cells (HMECs) that lack expression of ER (64, 65). This is in contrast to the mouse where PR^+^ luminal cells are CD29^low^, CD49f^low^, CD24^+^, and also have ER expression (59). In the canine mammary, tumor cell line (CMT U229) CD49f^+^, CD24^low^, CD44^+^ cells have been identified as tentative stem-like cells (66), with unknown ER/PR status.

Most data on hormone receptor status in the literature come from human mature luminal cells that mostly express ER and PR and mediate the proliferative effects of steroid hormones by paracrine signaling. However, E_2_ and P_4_ treatment also increase the MaSC population and in diestrus, when P_4_ levels are the highest, the number of CD24^+^/CD29^hi^ mammary repopulating units increases 14 times in mice. Thus, steroids do influence stem cells, in both their proliferative and self-renewal abilities (52, 67). Oakes et al. suggest that a paracrine mediator is responsible for the proliferation and maintenance of human MaSCs, and a likely candidate is P_4_-regulated RANKL (3).

The PR has two modes of mitogenic action in the mouse mammary gland; a cyclin D1-dependent stimulation of proliferation in PR^+^ cells and a RANKL-mediated paracrine action on nearby PR^−^ mammary epithelial cells (68). Steroid hormone-receptor positive cells release RANKL to neighboring stem cells in a paracrine manner. In a neighboring PR-negative luminal progenitor cell, RANKL can stimulate the transcription factor E74-like factor 5 and thus promote alveolar development (69). P_4_ also drives the CD24^+^/CD29^lo^/CD61^+^ luminal progenitor cell population to a CD24^+^/CD29^lo^/CD61^−^ mature luminal cell differentiation, resulting in mature alveolar lineage expansion. Another possible mediator of paracrine signaling is neuregulin (Nrg1), which is produced in the basal epithelial cells and is a direct transcriptional target of tumor protein 63 (p63). P63 KO mice have several developmental defects such as no limbs, teeth, and mammary glands. Exogenous Nrg1 rescued the lactation through activation of human epidermal growth factor 4/Stat5 in neighboring mouse luminal epithelial cells (70). MaSCs not only receive signals in a paracrine manner indicating paracrine basal to luminal signaling but they can also signal back to regulate the luminal epithelium. This “teamwork” on breast development and homeostasis is only beginning to be unraveled (71).

## Growth Hormone

### GH in the Mammary Gland

Similar to steroid hormones P_4_ and E_2_, pituitary GH is also necessary for mammary gland development. GH deficiency impairs the mammary development in rats and mice. GH influences alveolar and duct development and limits side branching (72). In humans with Laron syndrome, a mutated GH receptor (GHR) affects mammary gland development. Patients with Laron syndrome are shorter, have no diabetes type II and their risk of getting all kinds of cancer is almost 0 (73). By contrast, patients with hypersecretion of GH (acromegaly) and individuals who are taller in height have a higher cancer incidence (18). A sustained exposure to steroid hormones is the best established risk factor for human breast cancer (74), and this also influences canine mammary gland proliferation and carcinogenesis (75, 76). Sustained exposure leads to nodules of lobular hyperplasia and simple or complex adenomas in the mammary gland (76). The proliferative effect on the mammary gland coincides with increased plasma GH concentrations. Dogs with endogenous high plasma P_4_ concentrations or treated with exogenous MPA were found to have high plasma GH concentrations. We observed that the canine mammary gland produces GH locally after MPA administration (77). As well as finding immune reactive GH in the mammary gland and a steep decrease in plasma GH concentrations after complete mastectomy, we found expression of GH mRNA in both the canine and feline mammary gland (78). Next, we demonstrated that in the dog, mammary expression of GH was initiated at the same start site as GH from the pituitary (79) but the pituitary-specific POU domain transcription factor was absent from mammary tissue. Analysis of the GH promoter revealed a putative PRE (80) but experiments using a GH-promoter luciferase construct did not show a direct transactivation by P_4_-activated PR in canine mammary tumor cell lines (81). In tumors, the staining intensity of the PR varied from no staining, normal nuclear staining to remarkable heterogeneous and perinuclear staining and cytoplasmic staining in spindle cells (82). The major pathways that were activated after prolonged MPA exposure in dogs were determined through gene expression studies. Both mammary tissue and cell lines were used to identify carcinoma-related expression profiles (83–85). Autocrine production of GH signals in human MCF7 cells *in vitro* are associated with a more invasive phenotype and *in vivo* with more aggressive tumors (86) indicating that GH activates the same pathways in humans as in dogs.

### GH Signaling

**Figure 3:**
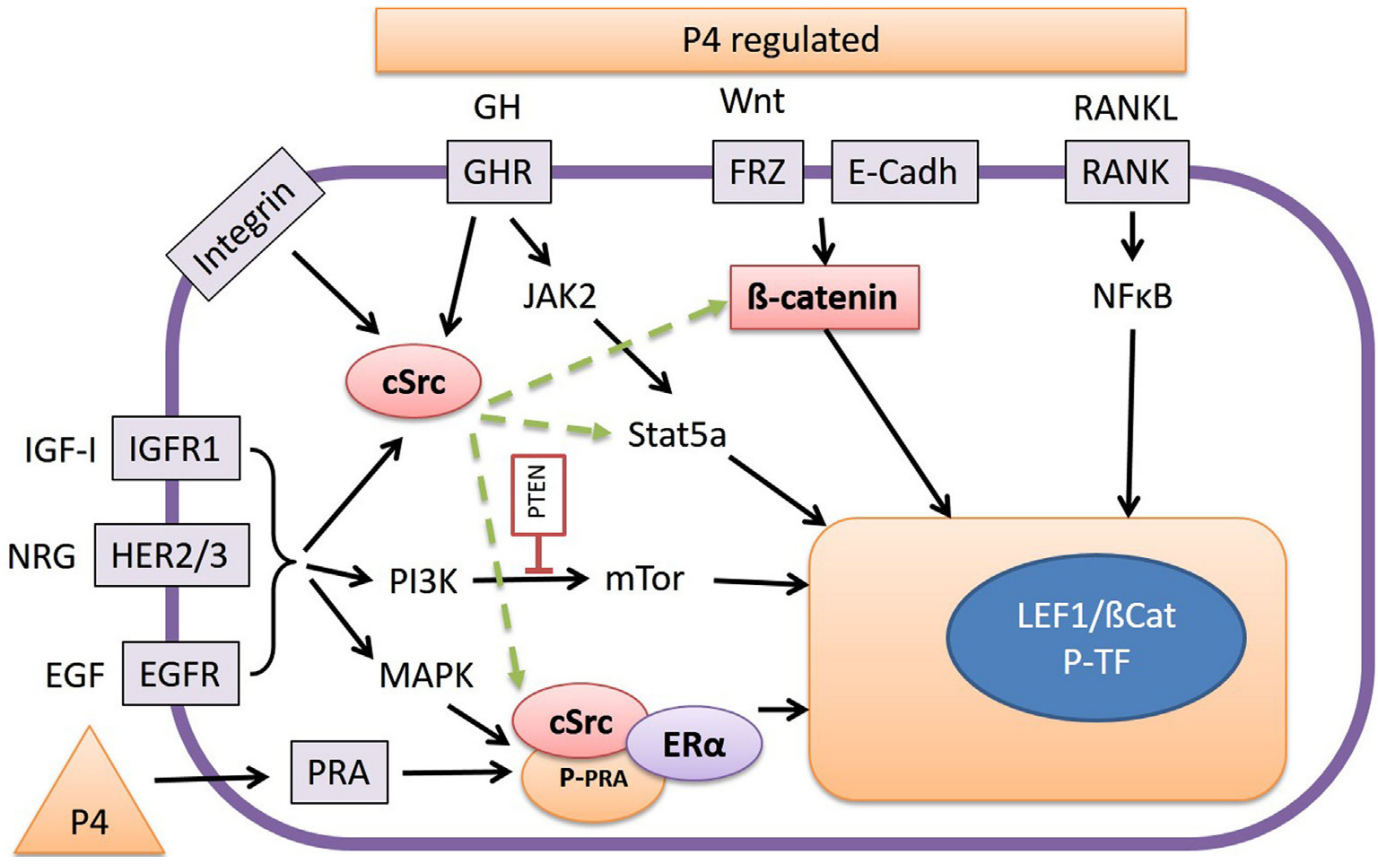
**The release of growth hormone (GH), Wnt, or RANKL, as stimulated by progesterone (P_4_), promotes interaction with various signal transduction pathways**. RANKL stimulates the NFκB pathway, and Wnt proteins stabilize cytoplasmic β-catenin, which under basal conditions is bound to E-Cadherin, and both activate Wnt target genes by forming a complex with T-cell transcription factors such as LEF-1. GH signals through the Janus kinase 2/Stat5a pathway but also by activation of cSrc. Inhibition of cSrc with the inhibitor Dasatinib results in inhibition of Wnt reporter activity in our canine cell lines by a yet unknown mechanism. As well as GH, integrins, type-1 IGF receptors (IGFR1), and the HER-family of tyrosine kinase receptors may also be involved in cSrc activation or stimulation of phosphatidyl-3-kinase or mitogen-activated protein kinase pathways. The high Wnt activity in our cell lines is associated with elevated expression of the HER receptors. Finally cSrc may also form a ternary complex with P_4_ receptor-A (PRA) and E_2_ receptor-α (ERα) resulting in activation/phosphorylation of nuclear transcription factors.

Growth hormone is a peptide hormone closely related to PRL that exerts its action through GHR present in the cell membrane as constitutively dimerized single transmembrane proteins (87). Upon binding of GH ligands, GHR dimers are transphosphorylated by JAK2 tyrosine kinases that result in activation of multiple downstream signaling pathways, including the STAT pathway, the MAPK pathway, the PI3K pathway, and the protein kinase C (PKC) pathway (88, 89) (Figure 3). Signaling through the JAK/Stat pathway is initiated by phosphorylation of Stat proteins by JAK2. Subsequent dissociation of Stat proteins from GHR is followed by their dimerization and translocation to the nucleus to activate target gene expression. Activation of Stat5A and B isoforms plays an important role in mammary gland development and results in transcriptional activation of multiple target genes including insulin-like growth factor 1 (IGF-1) and serum protease inhibitor 2.1 (spi2.1) (10, 90).

In the canine CMT-U27 cell line, a GHR-mediated growth effect was found as a consequence of increased cell survival by increased p-ERK 1/2 expression. This resulted in proliferation and an increased number of cells in the S and G2M phase (91). When GH binds to the GHR the tyrosine kinase JAK2 is activated, and this activated JAK2 phosphorylates various signaling mediators with the most important being p-ERK1/2 (MAPK). Activated MAPK signals *via* myelocytomatosis viral oncogene homolog (MYC) and c-AMP response element-binding protein (CREB) in the nucleus to activate the transcription machinery (91). This transcription machinery is also stimulated by the PI3K/protein kinase B (AKT) pathway where GH indirectly signals *via* IGF-1 enhancing the PI3K/mammalian target of rapamycin (mTOR) pathway (Figure 3).

### GH Signaling in Mammary Cancer

Expression of GH mRNA has also been observed in human breast cancer specimens (18, 92). This GH may interact with GHR-positive cells that are found in some 90% of human DCIS lesions and in 4–19% of normal breast epithelial cells (18). These GHR^+^ cells can form GH-dependent mammospheres, suggesting that GH may stimulate stem/progenitors to enter the cell cycle. The subpopulation of GHR^+^ cells also contains more progenitor cells with bipotent and myoepithelial differentiation potential compared to the GHR^−^ cell population (18). Mammosphere-initiating cells from both human and mouse mammary gland can repopulate the cleared mammary fat pad *in vivo* (93) and both the same mammospheres and T47D human cancer cells can be stimulated by P_4_ to produce GH. We also found this to be the case in mammary tissue of the dog (18, 82) indicating that there is a link in the mammary gland between P_4_ stimulation, GH secretion, and GH/GHR activation with GH having a paracrine role (Figure 2). The effect is most likely paracrine because GHR and PR cells differ in EpCam activity in humans. PR-positive cells are Lin^−^, EpCam^+^, CD49f^+^, ALDH^+^, and HER3^+^, whereas GHR-positive cells are Lin^−^, EpCam^+/−^, CD49f^high^, and ALDH^+^ (18, 59). EpCam^+^ cells are luminal progenitor cells and EpCam^−^ cells are stem and early progenitor cells (18, 59). There is also a discrepancy for GH and ALDH^+^ status. Only a minority of GH-producing cells were ALDH^+^ in a subset of HMECs, whereas 66% of the sorted ALDH^+^ cells were GHR^+^ and CD49^high^ (18). Cell sorting by fluorescence-activated cell sorting, however, is not always representative of the regenerative potential of the selected cells. Cell characteristics and stem cell origin can only be reliably gained from lineage tracing analysis. Using lineage trace experiments, Van Keymeulen demonstrated that the mammary gland in the mouse contains different types of long-lived stem cells that are derived from independent precursors during or prior to the onset of puberty (6). Thus, mammary gland stem cells can have a luminal or basal origin, both having a Lin^−^, CD24^+^, CD29^+^ phenotype (59, 60). For stem cell maintenance, the Wnt/β-catenin signaling can be used (60).

Growth hormone is mainly produced in the differentiated pituitary or placental cells but can also be produced locally within the mammary gland under the influence of P_4_. GH is responsible for the expansion of mammary stem and progenitor cells when the mammary gland grows, during puberty, pregnancy, and lactation and also in the menstrual cycle when P_4_ levels are higher. Development of the mammary gland is cumulatively effected by P_4_ and GH, and this regulatory growth process can eventually lead to changed ratios between progenitor and differentiated cells. Progenitor cells, being more proliferative, have a higher risk of oncogenic hits (18). This undesirable consequence of GH signaling in the mammary gland is not only mediated as a direct effect on GHR-positive cells in the MaSC compartment, but also in the stromal compartment, where indirect GH effects are mediated by the synthesis and release of insulin-like growth factor-I. The PR-positive cells within the mammary epithelium act as sensor cells for P_4_ signaling and stimulate the local production of not only GH but also RANKL and Wnt4 (17).

## Canonical Wnt Signaling

The Wnt signaling pathway is involved in regulation of several processes including cell proliferation, cell polarity, differentiation, and morphogenesis even from very earliest stages of embryogenesis (94). Wnt proteins, in general, are thought to signal through four distinct pathways; the so-called canonical or Wnt/β-catenin pathway and the non-canonical pathways where calcium (Ca^2+^) acts as a second messenger. These Wnt/Ca^2+^ pathways involve the PKC, the planar cell polarity (PCP) pathway involving Jun N-terminal kinase (JNK) and a pathway involving protein kinase A (PKA) that functions in muscle myogenesis (95). In the non-canonical Wnt/Ca^2+^ pathway, binding of Wnt to Frizzled receptor (Fzd) results in an increase of intracellular Ca^2+^ and the activation of Ca^2+^/calmodulin-dependent protein kinase II (CAMKII) and PKC, resulting in activated nuclear factors that turn on gene transcription. This Wnt/Ca^2+^ pathway can interact with the canonical Wnt pathway. CAMKII and PKC are able to phosphorylate β-catenin, thereby serving as a negative regulatory component of the canonical Wnt pathway (96–98). In the PCP pathway, the disheveled protein (DVL1) is recruited to the plasma membrane after binding of Wnt proteins to the Fzd. DVL1 activates small guanosine triphosphatases (GTPases), such as Ras homolog gene family member A (Rho-A) and cell division cycle 42. These GTPases activate Rho-associated kinase and JNK, leading to the transcription of target genes. This pathway functions also in regulation of cell movements and adherence (96). The third non-canonical pathway involves Fzd signaling *via* heterotrimeric guanosine triphosphate binding proteins (G proteins). G proteins activate phosphatidylinositol signaling *via* PKA and thus transcription activation (95).

The most important mediator of canonical Wnt signaling is β-catenin (Figure 3). In a cell lacking canonical Wnt activity, the vast majority of β-catenin protein is bound to E-cadherin at the cell membrane where it provides a link between the actin cytoskeleton and cell–cell junctions. The so-called β-catenin destruction complex rapidly degrades the remaining β-catenin in the cytoplasm. In this complex, proteins such as interacting protein (Axin1 and Axin2) and adenomatous polyposis coli (APC) act as scaffolds to bring β-catenin in association with casein kinase I (CK1) and glycogen synthase kinase 3 beta (GSK-3β), which phosphorylate β-catenin at Ser/Thr residues. This phosphorylated β-catenin is then targeted by the ubiquitination complex, resulting in subsequent proteosomal degradation (99). When the canonical Wnt pathway is activated through binding of Wnt ligands to Fzd and low density lipoprotein-related protein 5/6 (LRP5/6) coreceptors, the β-catenin destruction complex dissociates. As a consequence, β-catenin is dephosphorylated by protein phosphatase, resulting in its cytoplasmic stabilization. Stabilized β-catenin is then able to translocate to the nucleus where, in association with T-cell transcription factor/lymphoid enhancer-binding factor 1 transcription factors it regulates expression of target genes (100, 101). Recently, additional mediators of canonical Wnt signal have been identified that modulate stability of Fzd/LRP receptor complex on the cell membrane (i.e., Lgr5 as receptor with R-spondin ligand proteins), thereby enhancing the Wnt ligand signal (102). Experiments with antibodies show that these antibodies bind non-overlapping regions of LRP6 protein, suggesting that LRP6 contains separate binding sites for different classes of Wnt proteins (103). Axin binds preferably to the cytoplasmic tail of LRP6 that is phosphorylated through GSK3 and CK1γ (104). The Wnt-induced LRP6 phosphorylation brings Axin close to the Dvl protein, resulting in degradation of Axin by Dvl. This receptor protein phosphorylation therefore decreases the signal transduction pathway instead of amplifying it as would normally be expected (105). Wnt signaling appears to occur predominantly between cells that are close to each other, for example, in adult stem cell niches thus Wnt signals mediate close range signaling (106). To tightly regulate the canonical pathway activity, cells also express a number of Wnt antagonists, such as Dikkopf (Dkk) and secreted Frizzled-related protein (sFRP) that prevent Wnt proteins from binding to Fzd or LRP5/6. In addition, activation of the canonical Wnt pathway provides a negative feedback through stimulation of Axin2 expression (104, 107, 108).

### Wnt Signaling in Mammary Cancer

In the mammary gland, canonical Wnt activity is essential for both embryonic and postnatal development (109). During puberty and pregnancy, Wnt activity has been linked to P_4_ signaling. P_4_ has been shown to promote Wnt ligand expression (especially Wnt4) and to activate the downstream signaling in human, mice, and dogs (110, 111). The relevance of Wnt4 is confirmed by the phenotype of conditional knockouts of mammary Wnt4 expression in mice that have impaired ductal side branching, while overexpression of stabilized β-catenin in luminal epithelium results in precocious lobulo-alveolar development, alveologenesis, and neoplasia (112). In addition to its role in normal mammary gland development, deregulation of the canonical Wnt pathway is often associated with tumorigenesis (101). Oncogenic properties of Wnt proteins were first evident in the mammary gland, as Wnt1 and Wnt3 ligand were initially identified as insertion sites for a MMTV (113). Moreover, in human breast cancer, around 60% of examined clinical samples were shown to have elevated levels of nuclear and/or cytoplasmic β-catenin, suggesting an active signaling (114). In addition, in dogs exposed to prolonged MPA, *in vivo* strong upregulation of Wnt4 mRNA is also found. In dogs with spontaneous mammary carcinomas, we found a 6.8-fold induction of Wnt7a and also 2- to 3-fold changes in Wnt3, 4, 5a, and 5b mRNA (115). In both cases Wnt target genes such as cyclin D1, survivin, axin2, and cMyc were induced. In general, the same common activated pathways were found in canine carcinomas when compared to published human and mouse data (106, 116).

In most human tumors, constitutive activity of the canonical Wnt pathway was shown to be a consequence of mutations in APC or β-catenin (101). By contrast, such mutations are found only rarely in breast cancer (117). Rather, alternative explanations for the canonical pathway activation have been proposed for mammary tumors, including (1) mutations in other components of the pathway, (2) overexpression of Wnt ligands and other activators, (3) loss or downregulation of the antagonists, such as sFRP1, and (4) cross regulation by other deregulated pathways, such as epidermal growth factor (EGF), phosphatase and tensin homolog (PTEN), or tumor protein 53 (p53) signaling (117, 118). Information about canonical Wnt activity in canine mammary tumors is limited. Deregulation of the pathway has been proposed based on elevated β-catenin immunostaining and pathway analysis associated with molecular profiling of normal and tumorous tissue (19, 111, 119–121). None of the studies have, however, quantitatively assessed the activation of canonical Wnt signaling in canine mammary tumors or the underlying mechanism of its activation.

Wnt drives the formation of cells with CSC properties by regulating the expansion and proliferation of MaSCs (60, 122) and Wnt/β-catenin contribute to tumor progression and metastasis and related to all molecular subtypes of invasive breast cancer with a poor clinical outcome (123). The Wnt pathway is therefore a critical component in breast cancer development and a possible therapeutic target across cancer sub types. Wntless (WLS) appears to be required for the release of all Wnt proteins in both the canonical and non-canonical Wnt pathways. WLS is highly overexpressed in 48% in all subtypes of human breast cancer cases, and there is a strong correlation to HER2 overexpression (124). Together with upregulated Wnt signaling, our canine cell lines also have upregulated HER signaling (125). It remains to be shown whether the human and canine high Wnt- and HER-expressing cells point to a comparable stem cell.

## HER and the Interaction of Pathways

**Figure 4:**
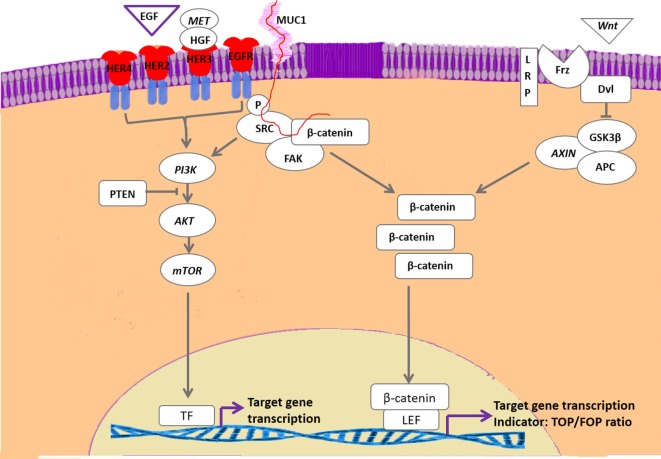
**Interaction of HER-activated pathways with canonical Wnt signaling**. With the exception of human epidermal growth factor 2, the family of epidermal growth factor receptor-related receptors is activated by epidermal growth factor (EGF) or EGF-related proteins as well as by cMet/HGF. The activated tyrosine kinase activity results in activation of the phosphatidyl-3-kinase (PI3K)/mammalian target of rapamycin (mTOR) pathway that is downregulated by PTEN activity. Inactivating PTEN mutations are frequently found in mammary carcinomas. The HER pathway may also phosphorylate and activate Src, resulting in stabilization of cytoplasmic β-catenin. Activation of the Wnt pathway also results in stabilization of cytoplasmic β-catenin that stimulates gene transcription by forming a complex with T-cell transcription factors such as lymphoid enhancer-binding factor 1. Both pathways are associated with development of therapy resistance. In our cell lines with high basal Wnt activity, inhibition of the PI3K/mTOR resulted in further increased Wnt activity, whereas SRC inhibition strongly inhibited Wnt activity.

As previously stated, we found a remarkable association between high intrinsic Wnt activity and high mRNA expression of the four members of the epidermal growth factor receptor (EGFR) family (126) of membrane receptor tyrosine kinases, commonly referred to as ErbB(1–4) or HER(1–4). A main pathway that is activated by the HER family is the PI3K pathway (Figure 4). Activated PI3K leads to phosphorylation of phosphatidylinositol 4,5 bisphosphate to phosphatidylinositol 3,4,4-triphosphate (PIP3). PIP3 is an important transducer of activating downstream components, such as AKT, which is then able to phosphorylate other targets, including the mTOR complex, a key regulator of cell growth, proliferation, survival, and protein synthesis. The mTOR pathway is frequently upregulated in breast cancer specimens. The pathway is stimulated by activation of HER signaling or by the type-1 IGF1-R. The HER2 receptor lacks a ligand-binding domain, whereas HER3 lacks tyrosine kinase activity. Nevertheless, HER2/3 heterodimers are among the strongest activators of the mTOR pathway (127, 128). PI3K/mTOR hyperactivation is linked to resistance to endocrine therapy of breast cancer in humans and dogs, resulting in tumor recurrence (129, 130). Dogs and humans have similarities in breast cancer tumor types and in the distribution of the tumor types. Approximately two-thirds of human breast cancers are steroid hormone receptor (ER/PR) positive and treated with combinations of selective estrogen receptor modulators, gonadotropin-releasing hormone agonists, and/or aromatase inhibitors (131–133), either alone or in combination with third-generation cytotoxic or biological therapies (134, 135). Of the hormone-receptor negative tumors, some 20% are characterized by HER2 amplification and overexpression leading to a dependency on the family of EGF or related growth factors. These breast cancers are treated with various HER2 inhibitors, both therapeutic antibodies and specific tyrosine kinase inhibitors (136–138). HER2 is a truncated receptor that does not bind ligands but has an active tyrosine kinase domain. Homodimers of HER2 cause weak signaling that is greatly enhanced after dimerization with HER1, 3, or 4 (139). In particular, after dimerization with HER3, which binds ligand but has no intrinsic kinase activity, the HER2/HER3 heterodimer is able to stimulate breast tumor cell proliferation (140, 141). The HER2 receptor is stabilized by heat-shock protein 90, but this also limits the capacity of HER2 to recruit HER3 to an active dimer (142). Activated HER3 receptors stimulate the MAPK and PI3K/AKT/mTOR signal transduction pathways (143). The latter is antagonized by PTEN, which dephosphorylates phosphoinositides generated by PI3K and also dephosphorylates focal adhesion kinase (FAK) and thus inhibits cell migration and integrin-mediated cell spreading. Loss of PTEN function by mutation or epigenetic silencing has been found frequently in various cancers including breast cancers (144). Severe PTEN deficiency has been associated with resistance to anti-HER2 therapy but confers susceptibility to inhibitors of the PI3K/AKT/mTOR pathway (145–147). Activation of the PI3K/AKT/mTOR pathway has been associated with resistance to hormone therapy that may be restored using selective mTOR inhibitors as Everolimus (148, 149). Everolimus therapy has recently commenced in human patients with ER^+^ breast cancer (150). Studies have shown that hormonal therapy combined of Everolimus leads to an increased survival (151, 152). In our *in vitro* studies with canine cell lines with high basal Wnt activity, a possible negative side effect of Everolimus has been found. Unexpectedly, inhibition of the PI3K/mTOR pathway with Everolimus, or a dual PI3K/mTOR inhibitor, stimulated the Wnt activity measured by reporter constructs. So, although Everolimus inhibited proliferation of the canine mammary cancer cells, it stimulated Wnt activity and thereby potentially their metastatic capacity and recruitment of stem cells. Recently, a new model of EGF receptor signaling in mammary cells was presented. This confirmed the involvement of MAPK, PI3K/AKT/mTOR, and STAT pathways, but due to complex interactions the involvement of cSrc, which is often overexpressed along with the HERs, remained unclear (153).

Apart from activation of MAPK and PI3K pathways, it has been proposed that HER dimers may also transduce signals through cSrc/FAK complexes (154). These complexes interact with the extracellular matrix, cell migration signals, integrins, mucin 1 (Muc1), and β-catenin *via* the Wnt pathway. The Wnt activity decreases when our cells are treated with a FAK inhibitor (125). This Focal-adhesion kinase canonical pathway is also related to resistance to estrogen deprivation and cSrc in ER^+^ breast cancer. Dasatinib, a pan-Src inhibitor, has shown a mixed success in clinical trials of ER^+^ patients. Recently, experiments in human MCF-7 cells, modeling resistance to aromatase inhibitors and tamoxifen showed that dasatinib plus endocrine therapy gave a dose-dependent decrease in proliferation and re-sensitized them to the endocrine therapy. Dasatinib also caused an inhibition of the AKT and ERK1/2 downstream pathway and inhibition of cSrc also showed a decrease in cell migration. These data suggest that cSrc produced the endocrine resistant cell in different ways (155). In our canine carcinoma cell lines with a highly upregulated Wnt signaling, we also found a decrease in cell proliferation when we treated these cells with a cSrc inhibitor but more importantly we found that the upregulated Wnt signaling that followed Everolimus treatment was dose-dependently reduced (125) (Figure 4). The fact that breast cancer tumors become therapy resistant is still the most important problem in breast cancer treatment. Major factors associated with resistance are (1) overexpression of EGFR, HER2, insulin-like growth factor 1 receptor (2), loss of ER expression (3), changes in extracellular matrix (4), mutations in PI3K (PTEN) or MAPK pathways, and (5) EMT and CSC processes (Wnt stimulated). However, clinical trials with inhibition of EGF/IGF signaling or PI3K activity are either suboptimal or even disappointing with respect to inhibition of breast cancer progression (156–161). Renoir et al. showed the relation of breast cancer and extra nuclear ERα with PI3K and cSrc. ERα forms complexes with PI3K and cSrc making these pathways potential targets for therapeutic intervention (162).

## Conclusion

The heterogeneity of the mammary gland and various breast cancer tumor subtypes make this disease hard to predict and to treat. There is no single-standard treatment, and no way to determine which tumor will respond to therapy even when a predictive biomarker is present. Because the mouse has a different lobular alveolar structure and cannot form spontaneous breast tumors the dog may be an attractive model to study hormone dependence, Wnt and GH signaling. The GH, P_4_, and Wnt pathways are major players in the development of mammary gland tumors, and this review shows that there are important key players between these pathways and others such as HER overexpression. *In vitro* studies in canine mammary cancer cell lines show that cSrc influences these pathways and so combination therapies of cSrc and mTOR inhibitors, or direct targeting of P_4_ or GH signaling are therefore possible new targets for therapeutic interventions.

## Author Contributions

ET-S, AG, and JM wrote and approved the final review.

## Conflict of Interest Statement

The authors declare that the research was conducted in the absence of any commercial or financial relationships that could be construed as a potential conflict of interest.
